# Replacement of stacked transgenes *in planta*

**DOI:** 10.1111/pbi.13172

**Published:** 2019-06-12

**Authors:** Weiqiang Chen, Gurminder Kaur, Lili Hou, Ruyu Li, David W. Ow

**Affiliations:** ^1^ Plant Gene Engineering Center Chinese Academy of Sciences Key Laboratory of South China Agricultural Plant Molecular Analysis and Genetic Improvement Guangdong Key Laboratory of Applied Botany South China Botanical Garden Guangzhou China; ^2^ University of Chinese Academy of Sciences Beijing China

**Keywords:** gene stacking, transgene replacement, GMO, recombinase, Bxb1, Cre

While GM crops of past years contain one to few traits, the continuous discovery of new trait genes would mean that over time, crops could end up with a large number of transgene insertions. If they were dispersed throughout the genome, extensive breeding would be needed to reassemble all of them into a single breeding line. Stacking new DNA to a preexisting transgenic locus insures that the package of transgenes can be transmitted through breeding programmes as a single locus rather than as segregating loci. Several versions of recombinase‐mediated gene stacking have been described (Srivastava and Thomson, 2016), and we previously reported on an *in planta* gene stacking method using the Bxb1 integrase for site‐specific integration followed by the Cre recombinase for removal of unneeded DNA (Hou *et al*., 2014). The described method permits the sequential addition of transgenes as each integrating molecule brings a new recombination target for the next round of integration. However, should a need arises later on that requires removal of existing transgenic DNA, one possibility would be to use sequence‐specific nucleases such as zinc‐finger nucleases, TALEN or CRISPR/Cas9 (Murovec *et al*., 2017; Weeks *et al*., 2016) to cut at specific targets and induce host‐mediated repair through non‐homologous end joining or homologous recombination. Alternatively, as we show here, it is possible to delete or replace preexisting transgenes by the same Bxb1/Cre recombinase‐mediated gene stacking system. Moreover, commercial crop improvement using this gene deletion/replacement strategy has freedom to operate, as opposed to patent licences needed for use of sequence‐specific nuclease‐based tools (Chen and Ow, 2017).

We had previously reported stacking two rounds of transgenes into a tobacco target line (Hou *et al*., 2014) that led to creation of line 23.C.4‐9.d8.BC1 with the structure depicted in Figure 1a. To test a gene replacement strategy, microparticle bombardment (Li *et al*., 2016) was used to deliver pHL002 (Figure 1b) along with a Bxb1 integrase‐expressing construct (not shown) into leaf explants of line 23.C.4‐9.d8.BC1. This integrating molecule differs slightly from the vectors described previously in that there is an additional *lox* site situated between the *npt*‐distal *attB* and the trait gene exemplified by *OsO3L2‐2B*. *OsO3L2‐2B* is a rice gene that has been shown to lower rice cadmium accumulation (Wang *et al*., 2016, 2019), and here, it serves as a DNA fragment to replace the previously stacked transgenes. Bxb1‐mediated site‐specific integration of pHL002 inserts *OsO3L2‐2B* into the target locus placing directly oriented *lox* sites to flank the previously stacked transgenes *gus*,* luc* and *gfp* (Figure 1c). This should permit the subsequent deletion of the previously stacked transgenes to produce the configurations shown in Figure 1d or e depending on inversion of the DNA bound by the outermost *lox* sites.

**Figure 1:**
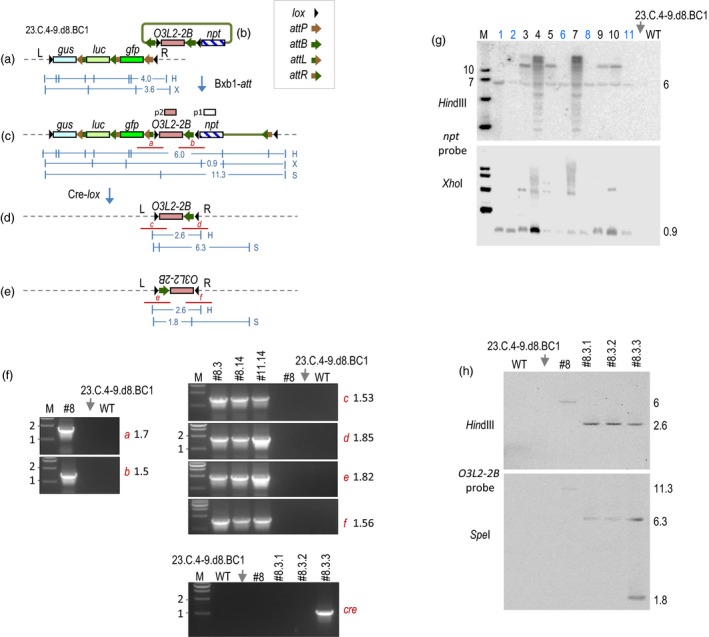
Bxb1‐mediated gene replacement. Tobacco stacked line 23.C.4‐9.d8.BC1 (Hou *et al*., 2014) with structure shown in (a) harbours an *attP* site for the integration by pHL002 (b) mediated by the co‐introduction of Bxb1 recombinase‐expressing pC35S‐BNK (not shown, Yau *et al*., 2011). Recombination with the *npt*‐distal *attB* yields the configuration shown in (c). Configuration from recombination with the *npt*‐proximal *attB* not shown. Cre‐*lox* reaction deletes DNA between *lox* sites to yield the structure shown in (d) or inverts DNA between oppositely oriented *lox* sites to yield the structure in (e). (f) Representative PCR detection of junctions *a*,* b*,* c*,* d, e, f* and *cre* gene. (g) Representative Southern blots of regenerated plants probed with *npt* DNA (p1) show a 6 kb *Hin*dIII band spanning from *gfp* to *npt* DNA and a 0.9 kb *Xho*I *npt* specific band, but only lines 1, 2, 6, 8 and 11 (blue lettering) show them as the only hybridizing band. Other lines with additional bands indicate additional copies integrated elsewhere in the genome. (h) Southern blot of F2 plants #8.3.1, #8.3.2 and #8.3.3 with *O3L2‐2B* probe (p2) detects a 2.6 kb *Hin*dIII band and a 6.3 kb *Spe*I band, as well as a 1.8 kb *Spe*I band in #8.3.3. Symbols for recombination sites as indicated. *gus*: beta‐glucuronidase gene, *luc*: firefly luciferase gene, *gfp*: green fluorescent protein gene, *OsO3L2*: rice *O3L2* gene 2B fragment, *npt*: neomycin phosphotransferase gene. L and R: T‐DNA left and right borders. *Hin*dlll (H), *Xho*l (X) and *Spe*l (S) sites and expected sizes (kb) of DNA fragments shown in blue. Red lines show PCR‐detected recombination junctions. M is marker lane, fragment sizes in kb. Gene promoters and terminators not shown; all genes transcribe from left to right except for inverted *O3L2‐2B* in (e). Protocol for biolistic site‐specific integration described in Li *et al*. (2016).

Out of ~200 shoots (due to *npt*) regenerated on kanamycin‐containing plates, a first PCR analysis detected 60 shoots positive for the *npt* DNA. A second PCR analysis for the expected integration junctions (Figure 1c) found only 11 plants with the correct junctions *a* and *b* (~1.7 and ~1.5 kb, respectively). The rest of the shoots could represent random or imperfect insertions of pHL002. After cloning into a vector for sequencing, the PCR products from all 11 plants confirmed the expected left and right junction sequences.

Southern hybridization with an *npt* probe (p1) detected a 6.0 kb *Hin*dIII band and 0.9 kb *Xho*I band in the genomic DNA from each of the 11 integrants (Figure 1g), and sizes of these bands were consistent with site‐specific integration. For the genomic DNA from WT (wild type) or the parental stacked line 23.C.4‐9.d8.BC1, hybridization was not found. As there was no other *Xho*I or *Hin*dIII band detected in integrant lines #1, #2, #6, #8 and #11, these five plants most likely lack additional copies of pHL002 elsewhere in the genome.

To remove the *lox*‐flanked DNA, we took hemizygous lines #2, #8, #11 to pollinate a homozygous *cre*‐expression line where *cre* is expressed from the CaMV 35S RNA promoter (Hou *et al*., 2014). A total of 78 F1 seedlings were genotyped by PCR to detect *OsO3L2‐2B* and *cre* DNA. The *cre* gene was detected in all 78 F1 seedlings as expected for a homozygous recipient. However, *OsO3L2‐2B* was detected in only 18 F1 individuals, which is lower than the 50% expected for Mendelian segregation. This may be caused by inefficient pollination or false negatives from difficulty in amplifying the *OsO3L2‐2B* gene.

Due an incidence of contamination, only 12 of the 18 seedlings survived for further analysis. PCR was used to detect the expected deletion‐specific recombination junctions *c* and *d* (Figure 1d), or if an inversion also took place, junctions *e* and *f* (Figure 1e). Recombination leading to inversions without deletions would yield only the parental *a* and *b* junctions. Of the 12 plants, junctions *c*,* d*,* e* and *f* were detected from #8.3, #8.14 and #11.14 (Figure 1f), while junctions *d*,* e* and *f* were found in #2.1. Junction *c* was found in one plant, and junction *d* in another, but for the remaining five plants, new junctions were not found.

However, for plants #8.3, #8.14 and #11.14*, gus* and *gfp* were also detected to indicate incomplete deletion (data not shown), which means that the F1 plants must be chimeric for the desired recombination. Along with the need to obtain germinal transmission of the deletion event, we took plants #8.3, #8.14 and #11.14 to the F2 generation. From the PCR analysis on 148 F2 seedlings (88 from #8.3, 48 from #8.14 and 12 from #11.14), only three seedlings from #8.3 (#8.3.1, #8.3.2 and #8.3.3) showed a PCR pattern lacking *luc* and *gus,* while having *OsO3L2‐2B*, and junctions *c* and *d*. Additionally, plant #8.3.3 also showed PCR products *e* and *f*, which could indicate that #8.3.3 harbours both the inverted and the noninverted transgene structure. This might mean that *cre* is still active, and indeed, a PCR could amplify the *cre* coding region in #8.3.3 but not in #8.3.1, #8.3.2 or in parental lines. (Figure 1f).

Southern blotting of the F2 plants #8.3.1, #8.3.2 and #8.3.3 showed that the *O3L2‐2B* probe (p2) hybridized a 2.6 kb *Hin*dIII band in all three F2 plants (Figure 1h). Since the *Hin*dIII sites are located outside of the inverted *lox* sites, the 2.6 kb *Hin*dIII band should be common for both transgene orientations. However, when cleaved with *Spe*I, which cuts within *O3L2‐2B*, the p2 probe detected a 6.3 kb band in all three F2 plants indicating the orientation shown in Figure 1d, while it also detected a 1.8 kb band in plant #8.3.3 indicating the inverse orientation in Figure 1e. This confirms that plant #8.3.3 harbours both the inverted and the noninverted copy. Since *cre* is still present in #8.3.3, Cre‐mediated inversion may be an ongoing reaction and is consistent with the relative abundance of the 6.3 kb and 1.8 kb bands. As for the control lanes, p2 hybridized to a 6.0 kb *Hin*dIII band and an 11.3 kb *Spe*I band in parental line #8, but to neither *Hin*dIII nor *Spe*I treated DNA from the WT or the parental stacked line 23.C.4‐9.d8.BC1.

To conclude, we demonstrate how one can use the same gene stacking steps of DNA site‐specific integration followed by removal of unneeded DNA to reconfigure a preexisting transgenic locus. Moreover, the new structure with a single attachment site (*attB* or *attP*) can continue to permit subsequent rounds of gene stacking or gene replacement. Whether this is used for deleting preexisting transgenes, replacing with new versions of the same gene or replacing with a completely new transgene, this transgene locus editing feature extends the flexibility of the Bxb1/Cre recombinase‐mediated system for transgene stacking.

## Conflict of interest

The authors declare no conflict of interests.

## Author Contributions

D.W.O designed the project. G.K., W.C., L.H. and R.L conducted the experiments. D.W.O., G.K. and W.C prepared the manuscript. All authors approved the final version of the manuscript.
